# Skim-Sequencing Reveals the Likely Origin of the Enigmatic Endangered Sunflower *Helianthus schweinitzii*

**DOI:** 10.3390/genes10121040

**Published:** 2019-12-15

**Authors:** Justin Anderson, Michael Kantar, Dan Bock, Kunsiri Chaw Grubbs, Edward Schilling, Loren Rieseberg

**Affiliations:** 1 Department of Tropical Plant and Soil Sciences, University of Hawaii, 102 St. John Plant Science Lab, 3190 Maile Way, Honolulu, HI 96822, USA; jeander1010@gmail.com; 2 Department of Organismic and Evolutionary Biology, Harvard University, Cambridge, MA 02138, USA; dan.g.bock@gmail.com; 3 Department of Biology, 202 Dalton Hall, Winthrop University, Rock Hill, SC 29733, USA; grubbsk@winthrop.edu; 4 Department of Ecology & Evolutionary Biology, University of Tennessee, Knoxville, TN 37996, USA; eschilling@utk.edu; 5 Biodiversity Research Centre and Department of Botany, University of British Columbia, 3529-6270 University Boulevard, Vancouver, BC V6T 1Z4, Canada; lriesebe@mail.ubc.ca

**Keywords:** polyploid origin, geographic distribution, crossing relationships, rDNA, cpDNA, Asteraceae

## Abstract

Resolving the origin of endangered taxa is an essential component of conservation. This information can be used to guide efforts of bolstering genetic diversity, and also enables species recovery and future evolutionary studies. Here, we used low-coverage whole genome sequencing to clarify the origin of *Helianthus schweinitzii*, an endangered tetraploid sunflower that is endemic to the Piedmont Plateau in the eastern United States. We surveyed four accessions representing four populations of *H. schweinitzii* and 38 accessions of six purported parental species. Using de novo approaches, we assembled 87,004 bp of the chloroplast genome and 6770 bp of the nuclear 35S rDNA. Phylogenetic reconstructions based on the chloroplast genome revealed no reciprocal monophyly of taxa. In contrast, nuclear rDNA data strongly supported the currently accepted sections of the genus *Helianthus*. Information from combined cpDNA and rDNA provided evidence that *H. schweinitzii* is likely an allo-tetraploid that formed as a result of hybridization between the diploids *Helianthus giganteus* and *Helianthus microcephalus*.

## 1. Introduction

Since the implementation of screens for allozyme variation [1,2] and through recent developments in next generation sequencing [3], molecular data have provided conservation managers and evolutionary biologists with key information for conservation planning. This information has been used, for example, to designate evolutionarily significant units for conservation action by augmenting knowledge on the morphology and ecology of populations [4,5]. As well, molecular data have enabled retrospective monitoring of effective population size and connectivity [6,7], and this has provided key information for translocation efforts, such as the identification of adaptive genetic variation or pathogens [3]. Lastly, genetic marker data have been instrumental in understanding and managing the destructive and constructive consequences of hybridization for declining populations. Destructive effects include outbreeding depression that may follow, for example, the introduction of invasive species and the formation of hybrid swarms [8]. Constructive effects include the reversal of inbreeding depression following from restricted population sizes [9], or the enhancement of adaptive potential [10]. 

Recently, interest has been mounting in using molecular data and knowledge of hybridization not only to boost the fitness of declining populations, but also to resurrect species that are already extinct. This can be achieved, for instance, through captive breeding programs in systems where the ancestry of extinct taxa only persists as hybrids [11]. Similarly, the identification of progenitor taxa for hybrid species could allow such information to be used, should the hybrid taxon go extinct. This strategy is likely to be of particular value in plants, for which a substantial fraction of speciation events involves hybridization [12]. Indeed, knowledge of parental species identity has been used with great success to re-synthesize hybrid species, with the goal of understanding the early stages of hybrid speciation, for both diploid [13,14] and polyploid taxa [15,16]. In this context, resolving the ancestry of endangered species is a topic of high conservation priority.

Next generation sequencing has enabled taxonomists and evolutionary biologists to resolve species boundaries even in systems that have historically been taxonomically challenging [17]. One implementation, referred to as genome skimming or ultra-barcoding, relies on sequencing whole genomes at low coverage, and assembling the high-copy organelle and rDNA fractions [18,19,20]. This approach has been applied to great effect for sorting previously unresolved phylogenies, and for identifying the diploid progenitor species of polyploid species [21,22,23]. 

Among plants, the *Helianthus* genus is well known for its taxonomic complexity. The recent origin of the group, multiple rounds of whole genome duplication [24], the large effective population size of taxa, and propensity to hybridize, are all factors that have made previous attempts of resolving species boundaries challenging [25,26]. Similar to the situation in other systems, next-generation sequencing is now being used to confirm previously established relationships, resolve ones that are still unknown, and classify newly described taxa [21,27,28]. 

**Figure 1:**
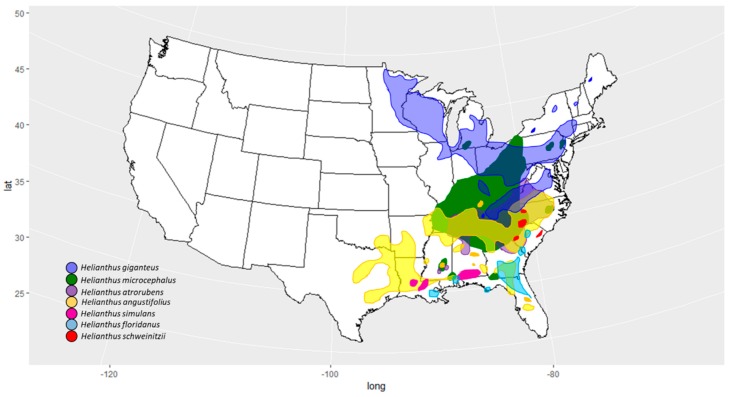
Species distributions of *Helianthus schweinitzii* and its potential parents (modified from Rogers et al., 1982 [36]).

One *Helianthus* species of particular conservation concern is *Helianthus schweinitzii* Torr. and A. Gray, a wild perennial endemic to the Piedmont Plateau of North and South Carolina (Figure 1). *Helianthus schweinitzii* is one of the rarest sunflowers in the United States and has been listed as endangered since 1991 [29]. Previous studies have revealed that the restricted distribution of *H. schweinitzii* is accompanied by limited genetic diversity [30]. While it is clear that the species has disomic inheritance [30,31], its classification has been problematic for taxonomists. Originally, *H. schweinitzii* was considered a hexaploid (2n = 6x = 102; [31]). Recently, it has been clarified that it is actually a tetraploid (2n = 4x = 68; [30,32]) whose genome size has expanded, potentially as a result of transposable element proliferation [33]. A hybrid origin for *H. schweinitzii* was first hypothesized in the 19th century by Torrey and Gray [34], but the parentage of the species has never been resolved. 

At least six perennial *Helianthus* species have been postulated as potential progenitors of *H. schweinitzii*, all of which have large geographic ranges, are phenotypically variable (plastic), and have a wide ability to hybridize (Figure 1; Table 1). These include *H. microcephalus, H. giganteus*, *H. angustifolius*, *H. simulans, H. atrorubens,* and *H. floridanus* [31]. These relationships have been postulated, in part, due to both the polyploidy nature of the plant and the range that overlaps with many different cross-compatible sunflowers (Figure 1; [31]).

The morphology within *Helianthus* has been extensively examined in an attempt to elucidate these inter-species relationships [26,31]. *Helianthus schweinitzii* is not a very distinctive plant, mostly identifiable by its small heads, dense pubescence, and thick tuberous roots [30,31]. Herbarium specimens have been confused with several different hybrids, including *H. microcephalus x H. giganteus* and *H. angustifolius x H. atrorubens* [31]. 

Three potential parents of *H. schweinitzii* are part of series *Angustifolii*: *H. angustifolius* (2n = 2x = 34)*, H. floridanus* (2n = 2x = 34)*,* and *H. simulans* (2n = 2x = 34). These are morphologically similar, have large regions of sympatry (Figure 1), and are cross compatible, hybridizing readily when they come into contact (Figure 1; Table 1; [26]). The other potential parents occur in separate series. *Helianthus giganteus*, part of series *Gigantei* (*H. giganteus,* 2n = 2x = 34), is morphologically diverse, widespread, and hybridizes with many other species across its range. *Helianthus microcephalus*, part of series *Microcephali* (*H. microcephalus*, 2n = 2x = 34) is distinguishable by its prolific small flower heads and shows a wide ability to hybridize (Table 1). *Helianthus atrorubens,* of series *Atrorubentes* (*H. atrorubens*, 2n = 2x = 34), is tall, has thick crowded basal leaves, and red florets with yellow or purple style branches. 

Perenniality, a trait occurring frequently in the *Helianthus* genus, may provide important clues regarding the parentage of *H. schweinitzii.* The perennial species within *Helianthus* show different modes of perennial habit including the formation of rhizomes, tubers, a deep taproot, or even re-growing from crown buds [31]. This variation is present in the potential parents of *H. schweinitzii¸* which itself has thick rhizomes and tuberous roots, which likely evolved as a response to periodic fires that once characterized its native habitat in the Carolina Piedmont [35]. *Helianthus giganteus* has large thick woody roots that can appear tuber-like and short rhizomes. *Helianthus microcephalus* has very fibrous roots, rhizomes and crown buds, but does not form tubers. *Helianthus angustifolius, H. floridanus* and *H. simulans* all have very fibrous roots and small slender rhizomes with many crown buds. *Helianthus atrorubens* contains poorly developed or absent rhizomes and regenerates from crown buds [36]. Thus, based on rhizome and root morphology, *H. schweinitzii* bears the closest resemblance to *H. giganteus*.

Many of the species examined here can be successfully crossed (Table 1). This characteristic has previously been exploited to study the evolutionary relationships of these perennial species [31,36]. However, hybrids often show reduced fertility and tend to not persist in nature [38]. Cytogenetic observations of perennial species show population-dependent pairing during meiosis [39]. While the crossing ability of many of the perennial sunflowers has been tested, there has been limited work exploring the cross-fertility of *H. schweinitzii,* due to the rarity of the plant. Differences in chromosomal structure between *H. schweinitzii* and other sunflowers are also not well characterized [30,40]. With these challenges in mind, the objective of this study was to identify the parental species of *H. schweinitzii*, which may be useful in conservation efforts. 

## 2. Materials and Methods

### 2.1. Plant Material, DNA Extraction, and Sequencing

The accessions of potential progenitor species used in this study were obtained from the United States Department of Agriculture (GRIN repository) and were chosen to maximize coverage of the geographical range for each species (Table 2). DNA was extracted from young leaf tissue using a Qiagen DNAeasy mini plant kit. *Helinathus schweinitzii* samples were collected under Department of Natural Resources collecting permit #32-2014 for the Rock Hill Blackjacks Heritage Preserve in Rock Hill, SC. Young leaves of live specimens that were in good health were sampled from Blackjacks Heritage Preserve/Wildlife management Area in Rock Hill, SC (https://www2.dnr.sc.gov/ManagedLands/ManagedLand/ManagedLand/46). The sites of these plants were recorded before the year 2000. Library preparation and DNA sequencing was conducted at Genome Quebec (McGill University and Genome Quebec Innovation Centre, QC, Canada). Individually-barcoded 100bp paired-end libraries were run on one lane of an Illumina HiSeq 2000 machine. Pooling of libraries was designed to achieve an even coverage for each species. Reads were trimmed to remove Illumina adapter contamination and low quality bases using Trimmomatic (v. 0.32; [41]). 

### 2.2. Assembly of Organelle and Nuclear DNA Regions

A total of 26,734,453,800 bases of sequence data were generated, averaging 581,183,778 bases per sample, which corresponds to circa 0.05X to 0.15X depth, depending on the sample’s genome size. Assembly of the chloroplast and mitochondrial genomes was attempted after first reducing the complexity of each library, to enrich for organelle genome reads. This was achieved by first aligning quality-filtered reads to the *H. annuus* chloroplast genome (GenBank accession NC007977) and mitochondrial genome (GenBank accession KF815390) using BWA-mem [42]. Reads that aligned to the chloroplast genome were assembled using VELVET [43]. We used a hash length of 21, and a minimum contig length of 100 bp. We also set a coverage cut-off of 10 reads. The resulting contigs were then ordered based on alignments to the corresponding organellar genome of *H. annuus*, and were merged using Geneious [44]. Regions not covered by Illumina reads, which led to low quality assemblies, were removed, leaving only the high-quality regions for analysis. Because few mitochondrial final assemblies were created due to low numbers of overlapping contigs, we discarded the mitochondrial genome from analyses and focused on the chloroplast genome for organelle DNA information. 

The nuclear 35S rDNA regions were assembled using a similar procedure. We relied on quality-filtered reads and used the VELVET de novo assembler [43], with the same parameters as used for the chloroplast DNA. Contigs for 35S rDNA were identified based on alignments to the corresponding *H. annuus* 35S reference (GenBank accession KF767534). A limitation of this is that only the most common SNP present in each individual is used. Additionally, 35S rDNA and chloroplast assemblies for six *H. giganteus* samples were obtained from Bock et al. (2014) [21]. These were generated using the same assembly pipeline described above. 

### 2.3. Phylogeny Reconstruction

The chloroplast and rDNA sequences were aligned using MAFFT [45] with default settings and were inspected and edited in Geneious by filtering low quality sequence [44]. Maximum likelihood (ML) trees were generated using PhyML [46] implemented in Geneious [44], with branch support estimated using the Shimodaira–Hasegawa-like (SH-like) procedure. Bayesian inference was conducted with MrBayes [47]. Briefly, the General-time-reversible (GTR) model was used to reconstruct the phylogeny. The Bayesian analysis used four runs, each with four Markov chains initiated from a random tree and run for 1,000,000 generations, which results in an Effective Sample Size of 336 for cpDNA and 936 for rDNA. The first 25% of all trees sampled before convergence were discarded as burn-in. Trees were rooted with *H. annuus* as the outgroup and reference genome source. For rDNA data, to further investigate the possibility that *H. schweinitzii* originated via repeated polyploidization events, we surveyed levels of sequence divergence among haplotypes obtained for each species. These analyses were based on the Tamura-Nei distance [48]. Series specific nucleotide variation was further explored in the assembled portion of the 35S rDNA data. Contigs were aligned to the *H. annuus* rDNA reference and SNPs were called using Geneious. Heterozygous (via overlapping contigs) and tri-allelic SNPs were removed. In total, 260 SNPs were called in the 35S rDNA between 200 bp and 6770 bp where all individuals had a full assembly. 

## 3. Results

**Figure 2:**
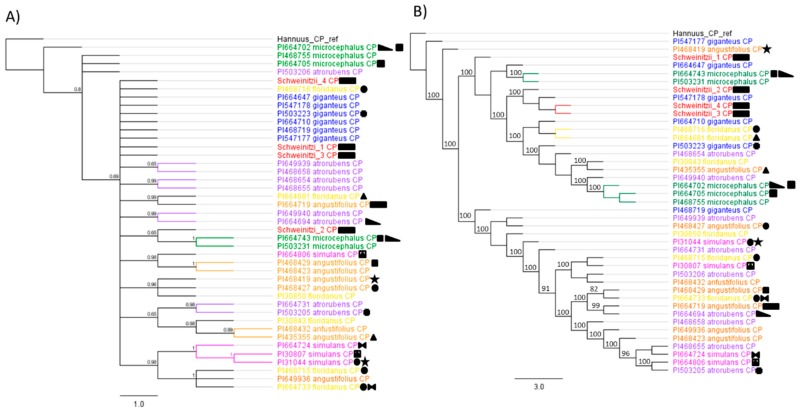
(**A**) Bayesian phylogenetic reconstruction of chloroplast phylogeny showing relationships of *Helianthus schweinitzii* and related species. (**B**) Maximum likelihood phylogenetic reconstruction of chloroplast phylogeny. Shared symbols indicate that accessions were collected within 100 km of each other. Support is shown for nodes with Shimodaira–Hasegawa-like (SH-like) values >70% and Bayesian posterior probabilities >0.7.

Phylogenetic analyses based on a chloroplast DNA alignment of 87,004 bp did not recover any perennial sunflower species as reciprocally monophyletic. Instead, many groupings tracked geography. These included, for example, accession pairs PI468716 (*H. floridanus*)—PI503223 (*H. giganteus*), and accession pairs PI31044 (*H. simulans*)—PI468715 (*H. floridanus*; Figure 1 and Figure 2; Table 2). While the branches were not monophyletic, there were some associations between *H. angustifolius*, *H. simulans* and *H. floridanus* of the *Angustifolii* series. *Helianthus schweinitzii* accessions repeatedly grouped with *H. microcephalus* and *H. giganteus* across analytical methods (Figure 2). Mitochondrial phylogenies were not informative due to limited coverage across taxa and poor alignments. 

**Figure 3:**
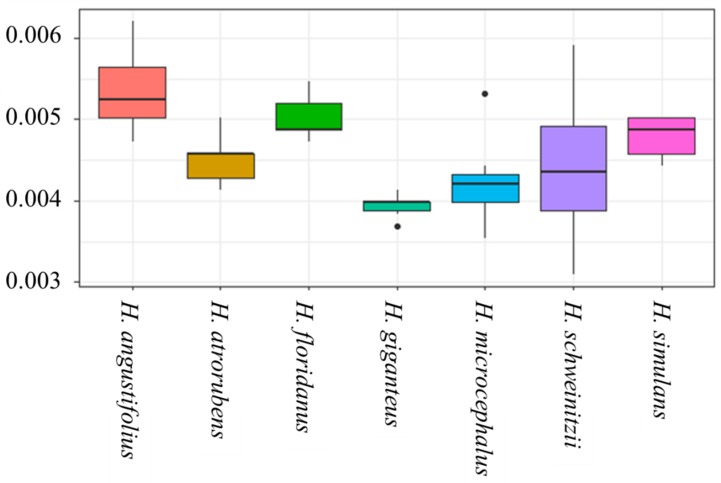
Box plots of sequence divergence calculated between individuals within species of *Helianthus* for partial rDNA haplotypes. Center lines show the medians; box limits indicate the 25th and 75th percentiles as determined by R software; whiskers extend 1.5 times the interquartile range from the 25th and 75th percentiles, outliers are represented by dots.

**Figure 4:**
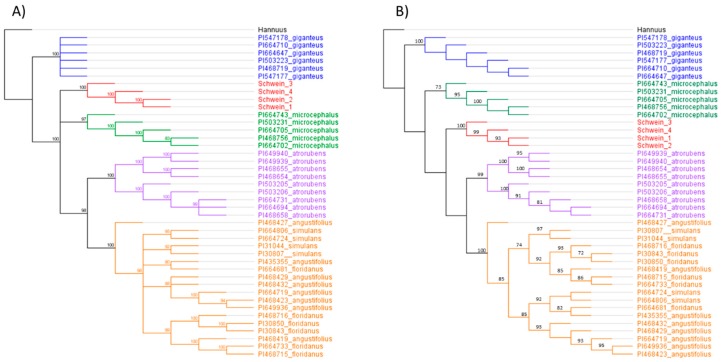
(**A**) Bayesian phylogenetic reconstruction of 35S rDNA showing relationships of *Helianthus schweinitzii* and related species; (**B**) Maximum likelihood phylogenetic reconstruction of 35S rDNA.

Analyses of rDNA sequence divergence revealed comparable levels of diversity for *H. schweinitzii* and candidate progenitor species (Figure 3). The level of sequence divergence between haplotypes was comparable between the diverse accessions of putative homoploid hybrids, which was higher than putative diploid parents, this agrees with previous expectations. Phylogenies based on the 35S rDNA alignment (6770 bp) revealed that many taxa form monophyletic groups, some with high support (Figure 4). Two major species groups were recovered. The first comprised *H. angustifolius, H. floridanus*, and *H. simulans*, with these species being polyphyletic. This is consistent with the idea previously advanced by Timme et al., 2007 [49], that *H. simulans* may be a homoploid hybrid of *H. angustifolius* and *H. floridanus*. The second group, *H. giganteus, H. atrorubens, H. microcephalus* and *H. schweinitzii* were recovered, all monophyletic. There was not strong phylogentic support for the association of *H. microcephalus* with *H. schweinitzii* despite the reported morphology-based characterization of *H. microcephalus* as a parental species [31]. 

The cpDNA tree did not recover monophyletic groups, but *H. schweinitzii* was consistently associated with *H. giganteus* and *H. microcephalus*. The rDNA tree did not identify the same associations as the cpDNA tree. This may be due to the different mode of inheritance of cp (maternal) and rDNA (biparental) which can cause differing tree topologies; this could be due to hybridization (chloroplast capture), insufficient sampling, and variable evolutionary rates.

## 4. Discussion

Chloroplast DNA variation can be used to explore species origin and, in the case of hybrid taxa, the direction of hybridization (i.e., the identity of the maternal progenitor). Also, the extent of polymorphism retained at the level of organelle DNA may be used to distinguish between the occurrence of single vs. multiple polyploid speciation events [50,51]. In this study, the chloroplast phylogeny did not recover any perennial *Helianthus* species as reciprocally monophyletic. This is in line with previous findings in other perennial *Helianthus* [21] as well as in annual *Helianthus* taxa [52]. These results can be explained by incomplete lineage sorting (ILS) or by reticulation. ILS, which is caused by retention of ancestral states, results in discordant phylogenetic relationships [53,54] and is likely common in sunflowers due to their recent radiation across North America [25,49,55,56]. In perennial taxa in particular, allelic coalescence may be delayed because these species are fewer generations removed from the speciation event, all else being equal. 

The alternative explanation, reticulation, results in systematic associations between species. These associations reflect historical organelle capture events occurring among pairs of taxa that are interfertile [56]. Previous results in annual *Helianthus* [52] have indicated that, relative to ILS, reticulation is likely more important in generating patterns of cytonuclear discordance such as those observed here. Indeed, we identified several cases of haplotype sharing among geographically proximate populations (Figure 2), which would indicate that hybridization is more likely than ILS. 

In the case of polyploid species, instances of limited or no chloroplast DNA variation have previously been interpreted as evidence for the occurrence of a single speciation event [50,51]. Cases where chloroplast DNA variation is extensive or comparable to that observed in candidate progenitors can be explained by two non-mutually exclusive scenarios, repeated polyploid speciation [57] or post-speciation reticulation. In the case of *H. schweinitzii*, the level of sequence divergence that we inferred among cpDNA haplotypes was similar for all perennial sunflowers. Therefore, because of the likely occurrence of reticulation and chloroplast capture in this system, our ability to infer the number of speciation events for *H. schweinitzii* is limited. The phylogenetic placement, based on the rDNA data, of *H. angustifolius*, *H. floridanus*, and *H. simulans*, is in agreement with previous taxonomic work. This is supported by the high level of cross fertility among these three species [31,36]. 

The interpretation of the cpDNA information is complicated because of ongoing ILS and introgression. However, *H. schweinitzii* shares more cpDNA haplotypes with *H. giganteus* than any of its other possible parents (Figure 2). The rDNA shows a trichotomy in the Bayesian inference, which includes *H. microcephalus and H. atrorubens,* while the maximum likelihood tree suggests a closer relationship with *H. atrorubens, H. angustifolius, H. simulans*, and *H. floridanus*, making it difficult to make definitive assessments. Thus, the most parsimonious explanation when considering cpDNA, rDNA, crossing data, and geography is with an allotetraploid origin from *H. microcephalus* and *H. giganteus*, as originally hypothesized by Heiser [31]. However, we are unable to fully exclude the possibility of an autopolyploid origin or that an extinct diploid is the progenitor species (or one of the progenitors), similar to the B genome in *Triticum* [58]. If *H. schweinitzii* was formed due to a hybrid origin, it is possible that *H. giganteus* served as maternal parent, while the paternal parent could be the extinct parent, perhaps the common ancestor of *H. atrorubens*, *H. angustifolius*, *H. simulans*, *and H. floridanus*. Based on the crossing studies, it is possible that bidirectional hybridization events lead to the origin of *H. schweinitzii*. The hypothesis of an extinct progenitor is also supported by the distinctive sesquiterpene lactone chemistry reported for *H. schweinitzii* [59] and the finding of Timme et al. [25] that polyploids formed their own clade in a 35S rDNA tree for *Helianthus*. However, it is important to keep in mind that novel secondary compounds are often generated in hybrids [60] and that concerted evolution among parental rDNA repeats (or the presence of both parental sites) in allopolyploids could create a convergent phylogenetic signal. Testing the hypothesis of an extinct progenitor will require additional genomic data. Another option would be to attempt to re-create *H. schweinitzii* from hybrids of *H. microcephalus* and *H. giganteus*, a previously reported successful cross [31]. 

## 5. Conclusions

The demonstration that *Helianthus schweinitzii* exhibits significant genetic distinctness from its progenitors heightens the need to conserve this distinctive but threatened species. In addition, the presence of well-formed tubers makes it of additional interest as a potential study system for tuber formation, as well as a possible source of genetic material for improvement of *H. tuberosus*. The two tuber-forming species of the genus are now proposed to have different sets of ancestors (*H. grosseserratus* and *H. hirsutus* for *H. tuberosus*, [21]; *H. giganteus* and *H. microcephalus* for *H. schweinitizii*, current study), increasing the likelihood that different sets of genes may be involved in tuber formation and chemistry in the two species. 

## Figures and Tables

**Table 1:** Crossing relationships among *H. schweinitzii* and its potential parents. Relationships are from Heiser et al., 1962 [37] and Rogers et al., 1982 [36], Y indicates a successful hybrid, N indicates no hybrid, and NA indicates no available information. Shading was used to avoid redundant information being presented in different parts of the table.

	*Helianthus atrorubens*	*Helianthus floridanus*	*Helianthus giganteus*	*Helianthus microcephalus*	*Helianthus simulans*	*Helianthus schweinitzii*
*H. angustifolius*	Y	Y	N	N	Y	Y
*H. atrorubens*		N	Y	Y	Y	N
*H. floridanus*			N	N	Y	N
*H. giganteus*				Y	N	Y
*H. microcephalus*					N	NA
*H. simulans*						NA

**Table 2:** Accession name and location of samples used in the study.

Accession	Species	Range	Latitude	Longitude
PI468419	*H. angustifolius*	East Central USA	29°39′0″N	82°19′0″W
PI435355	*H. angustifolius*	East Central USA	32°3′0″N	84°11′W
PI468423	*H. angustifolius*	East Central USA	33°32′N	92°28′0″W
PI468427	*H. angustifolius*	East Central USA	30°49′0″N	82°0′0″W
PI468429	*H. angustifolius*	East Central USA	34°18′0″N	79°2′W
PI468432	*H. angustifolius*	East Central USA	34°19′0″N	78°30′0″W
PI649936	*H. angustifolius*	East Central USA	35°23′57″N	86°1′0″W
PI664719	*H. angustifolius*	East Central USA	35°18′53″N	80°2′49″W
PI503206	*H. atrorubens*	East Central USA	37°0′0″N	77°0′0″W
PI649939	*H. atrorubens*	East Central USA	36°36′30″N	88°41′30″W
PI649940	*H. atrorubens*	East Central USA	33°53′26″N	86°49′33″W
PI664694	*H. atrorubens*	East Central USA	34°39′37″N	83°20′53″W
PI664731	*H. atrorubens*	East Central USA	33°11′40″N	79°31′32″W
PI503205	*H. atrorubens*	East Central USA	36°0′0″N	77°0′0″W
PI468654	*H. atrorubens*	East Central USA	33°1′0″N	84°42′0″W
PI468655	*H. atrorubens*	East Central USA	34°14′N	84°29′W
PI468658	*H. atrorubens*	East Central USA	33°49′0″N	81°6′0″W
PI664733	*H. floridanus*	South East USA	31°32′51″N	81°32′24″W
PI30843	*H. floridanus*	South East USA	29°42′53″N	85°1′31″W
PI30850	*H. floridanus*	South East USA	28°40′30″N	80°58′34″W
PI468715	*H. floridanus*	South East USA	30°33′0″N	81°49′0″W
PI468716	*H. floridanus*	South East USA	30°49′0″N	82°0′0″W
PI664681	*H. floridanus*	South East USA	31°18′17″N	83°48′40″W
PI547177	*H. giganteus*	East Central USA	46°37′00″N	90°46′00″W
PI664647	*H. giganteus*	East Central USA	41°35′27″N	83°45′43″W
PI664710	*H. giganteus*	East Central USA	35°48′42″N	82°11′50″W
PI468719	*H. giganteus*	East Central USA	36°18′00″N	78°35′00″W
PI547178	*H. giganteus*	East Central USA	45°15′00″N	88°36′00″W
PI503223	*H. giganteus*	East Central USA	36°00′00″N	77°00′00″W
PI664743	*H. microcephalus*	East Central USA	34°15′45″N	82°39′46″W
PI468756	*H. microcephalus*	East Central USA	36°7′0″N	79°25′0″W
PI503231	*H. microcephalus*	East Central USA	37°0′0″N	80°0′0″W
PI664702	*H. microcephalus*	East Central USA	34°56′51″N	83°5′21″W
PI664705	*H. microcephalus*	East Central USA	35°10′56″N	82°22′15″W
PI31044	*H. simulans*	South East USA	29°58′50″N	82°14′12″W
PI30807	*H. simulans*	South East USA	30°28′58″N	90°55′11″W
PI664724	*H. simulans*	South East USA	32°7′28″N	81°37′25″W
PI664806	*H. simulans*	South East USA	30°27′8″N	90°54′52″W
NA	*H. schweinitzii_01*	Piedmont plateau in North and South Carolina	34°54′7″N	81°1′18″W
NA	*H. schweinitzii_02*	Piedmont plateau in North and South Carolina	34°54′7″N	81°1′19″W
NA	*H. schweinitzii_03*	Piedmont plateau in North and South Carolina	34°56′29″N	81°0′28″W
NA	*H. schweinitzii_04*	Piedmont plateau in North and South Carolina	34°56′29″N	81°0′28″W
